# Non-Destructive Quantitative Characterization of Constituent Content in C/C–SiC Composites Based on Multispectral Photon-Counting X-Ray Detection

**DOI:** 10.3390/s26082331

**Published:** 2026-04-09

**Authors:** Xin Yan, Kai He, Guilong Gao, Jie Zhang, Yuetong Zhao, Gang Wang, Yiheng Liu, Xinlong Chang

**Affiliations:** 1 School of Missile Engineering, Rocket Force University of Engineering, Xi’an 710025, China; yanxin@opt.ac.cn (X.Y.); xinlongch_ch@163.com (X.C.); 2 State Key Laboratory of Ultrafast Optical Science and Technology, Xi’an Institute of Optics and Precision Mechanics, Chinese Academy of Sciences, Xi’an 710119, China; gaoguilong@opt.ac.cn (G.G.); zhangjie@opt.ac.cn (J.Z.); zhaoyuetong@opt.ac.cn (Y.Z.); wanggang@opt.ac.cn (G.W.); liuyiheng@opt.ac.cn (Y.L.)

**Keywords:** C/C–SiC composites, photon-counting detector (PCD), multispectral X-ray detection, non-destructive characterization, effective atomic number (*Z*_eff_), constituent inversion

## Abstract

**Highlights:**

**What are the main findings?**

- A physics-guided framework was developed for nondestructive quantitative characterization of constituent content in C/C–SiC composites using multispectral photon-counting X-ray detection.
- By jointly accounting for detector-response distortions and polychromatic beam-hardening effects, the method enables stable recovery of multispectral attenuation features and achieves SiC mass-fraction quantification with an error below 3 wt%.

**What are the implications of the main findings?**

- The proposed workflow extends photon-counting spectral correction from spectrum restoration to constituent-content inversion, providing a complete route from corrected attenuation features to quantitative composition characterization.
- This method provides a practical nondestructive paradigm for quality control and service evaluation of advanced ceramic-matrix composites, especially in material systems affected by thickness, density, and porosity variations.

**Abstract:**

To enable non-destructive quantitative characterization of constituent content in C/C–SiC ceramic-matrix composites, this study develops a physics-guided framework based on multispectral photon-counting X-ray detection. In practical photon-counting measurements, multispectral attenuation features are jointly distorted by detector-response non-idealities, including charge sharing, K-escape, and finite energy resolution, as well as by beam-hardening effects from the polychromatic X-ray source. To address this coupled problem, a Geant4 11.2-based detector-response model was incorporated into a unified correction workflow together with beam-hardening compensation, so that physically consistent multispectral attenuation vectors could be recovered for subsequent constituent inversion rather than merely for spectrum restoration. On this basis, a fine-grained theoretical database covering different SiC mass fractions was established, and quantitative constituent inversion was achieved by matching the corrected attenuation features to the database. Experimental results show that the proposed framework effectively suppresses thickness-dependent bias in attenuation measurements and yields an average relative error below 3% for pure aluminum. For C/C–SiC composites, the SiC mass fraction can be quantified with an accuracy better than 3 wt%. These results demonstrate that the proposed method provides a practical non-destructive route for constituent-content characterization in heterogeneous ceramic-matrix composites and is valuable for manufacturing quality control and in-service assessment.

## 1. Introduction

C/C–SiC (Carbon/Carbon–Silicon Carbide) ceramic matrix composites are high-performance materials in which carbon fibers reinforce a carbon matrix, while a SiC ceramic phase is introduced through a siliconization reaction. Owing to their low density, high specific strength, excellent high-temperature resistance, and oxidation resistance, C/C–SiC composites have been widely used in thermal protection systems for hypersonic vehicles and hot-section components of aero-engines, such as aero-engine hot-section components, spacecraft thermal protection systems, and critical parts like nose cones and wing leading edges for hypersonic aircraft [1,2,3,4,5]. However, the manufacturing of C/C–SiC composites typically involves a combination of complex processes, including Chemical Vapor Infiltration (CVI), Precursor Infiltration and Pyrolysis (PIP), and Reactive Melt Infiltration (RMI) [6]. This complexity leads to pronounced spatial inhomogeneity in the mass fractions of carbon fibers, carbon matrix, and SiC ceramic matrix within the material [7,8]. Furthermore, during long-term high-temperature service, the material undergoes degradation phenomena including localized oxidation, thermal stress-induced microcracking, and preferential depletion of the SiC phase, which further exacerbates the spatial distribution variance of constituent contents [9,10,11]. Studies have demonstrated that minor variations in the mass fraction of the SiC ceramic matrix directly affect the mechanical properties, thermophysical characteristics, and oxidation resistance of the material [7,12,13,14]. For instance, regions with insufficient SiC content are prone to becoming oxidation pathways, resulting in local failure and even overall structural damage. X-ray computed microtomography has also been employed to digitize fibrous C/C preforms and assess their structural/transport characteristics, illustrating the utility of X-ray-based nondestructive characterization for porous composites [15]. Therefore, achieving high-accuracy, nondestructive, and spatially resolved characterization of the constituent contents in C/C–SiC composites is of paramount engineering significance for manufacturing quality control, in-service condition assessment, and full life-cycle health management.

Currently, characterization methods for the constituent content of C/C–SiC composites primarily fall into two categories: destructive analysis and non-destructive testing (NDT). Destructive techniques, such as scanning electron microscopy (SEM) coupled with energy-dispersive spectroscopy (EDS), X-ray diffraction (XRD), and thermogravimetric analysis (TGA), provide high local accuracy [16]. However, they require destructive specimen preparation (e.g., cutting and polishing), making them unsuitable for in-service components and impractical for large-area statistical assessment. In contrast, non-destructive approaches including ultrasonic inspection, infrared thermography [17], terahertz time-domain spectroscopy (THz–TDS) [18], dynamic refractive index correction [19], and conventional X-ray absorption imaging [20] enable global or macroscopic defect evaluation [21,22], but their capability for quantitative composition characterization remains limited. Specifically, ultrasound is sensitive to density variations yet struggles to differentiate between carbon and SiC phases; conventional X-ray absorption provides only projection attenuation information at a single energy level, which is severely affected by beam-hardening effects [23,24] and thus cannot accurately invert multi-phase component proportions. In recent years, dual-energy X-ray CT has shown progress in separating composite density and effective atomic number ($Z_{eff}$) [25,26,27]. Nevertheless, constrained by the energy resolution of integrating detectors, issues such as incomplete beam-hardening correction and insufficient utilization of energy information persist [28].

Conventional dual-energy and multi-energy decomposition methods provide an important reference route for quantitative material characterization [29]. In two-component systems, attenuation measurements acquired in different energy windows can be used to estimate basis-material fractions and, in some cases, to further derive effective atomic number or other composition-sensitive parameters. Therefore, for a material system such as C/C–SiC, which can be approximately treated as a carbon-dominated phase together with a SiC ceramic phase, dual-energy and multi-energy analysis are directly relevant rather than conceptually unrelated to the present work [20].

However, practical photon-counting measurements differ from idealized decomposition scenarios in that the measured multi-bin attenuation data are jointly affected by beam hardening and detector-response non-idealities. Under polychromatic X-ray irradiation, beam hardening introduces thickness-dependent spectral bias, whereas charge sharing, K-escape, finite energy resolution, and related detector effects cause substantial count redistribution, especially in the low-energy bins [24,25,30]. As a result, attenuation features recovered from the measured data may still deviate from the true material-dependent response if only conventional decomposition or empirical beam-hardening correction is applied.

Recent studies on photon-counting spectral correction have further shown that the practical value of a detector-response correction scheme is usually established through quantitative performance evaluation rather than through the mathematical form of the response model alone [31]. For example, Jumanazarov et al. reported that spectral correction improved the estimation accuracy of electron density and effective atomic number by factors of 1.6 and 3.8, respectively, while maintaining well-conditioned correction matrices [32]. Taguchi et al. also showed that, compared with a simplified count-loss-based model, a more complete pulse-pileup-and-charge-sharing compensation model substantially reduced bias under high-count-loss conditions [33]. These studies indicate that the advantage of a more physically complete response formulation should be understood in terms of improved correction fidelity and downstream quantitative performance, rather than in terms of decomposition notation itself.

In this context, the present study is not centered on proposing a new universal DRM mathematical form. Instead, it extends physics-based photon-counting spectral correction toward a more task-specific objective, namely, nondestructive quantitative inversion of SiC mass fraction in C/C–SiC composites. To this end, a Geant4-based detector-response model is incorporated into a unified correction workflow together with beam-hardening compensation, so that physically consistent multispectral attenuation vectors can be recovered for subsequent inversion [34,35]. On this basis, a fine-grained theoretical database covering practical SiC mass-fraction ranges is constructed, and the corrected attenuation features are directly linked to constituent-content inversion through database matching. Therefore, the main contribution of this work lies in establishing a task-oriented and physically consistent route from distorted multispectral PCD measurements to quantitative constituent-content inversion in heterogeneous ceramic-matrix composites.

## 2. Theoretical Background and Methods

We addressed two major sources of systematic error—beam hardening and detector-response distortion—to establish the theoretical foundation for quantitative multi-energy characterization and to describe the proposed framework for joint correction and constituent-content inversion. The workflow comprises PCD physical-response modeling, response-matrix construction, synchronized joint correction through response restoration and beam-hardening compensation, development of a $Z_{eff}$—attenuation characteristic database, and, finally, constituent-content inversion and experimental validation.

### 2.1. Principles of Multi-Energy X-Ray Attenuation and Effective Atomic Number ($Z_{eff}$) Characterization

X-ray interactions with matter are primarily governed by the photoelectric effect, Compton (incoherent) scattering, and Rayleigh (coherent) scattering. Within the energy range commonly used for industrial non-destructive testing (20–200 keV), the photoelectric effect and Compton scattering dominate, whereas the contribution of Rayleigh scattering is relatively small and is often neglected. Notably, the photoelectric absorption cross-section exhibits a strong dependence on the atomic number of the material. For monochromatic X-rays, attenuation follows the Beer–Lambert law:

(1) $$I=I_{0}\exp\left(-\int \mu \left(E,r\right)\;dl\right)$$

where $I_{0}$ and I denote the incident and transmitted X-ray intensities, respectively; μ(E,r) is the linear attenuation coefficient at position r for photon energy E; and dl represents an infinitesimal path element along the ray trajectory. For polychromatic X-rays with a continuous spectrum, the detected intensity is given by the energy integral of contributions from photons at different energies.

**Figure 1:**
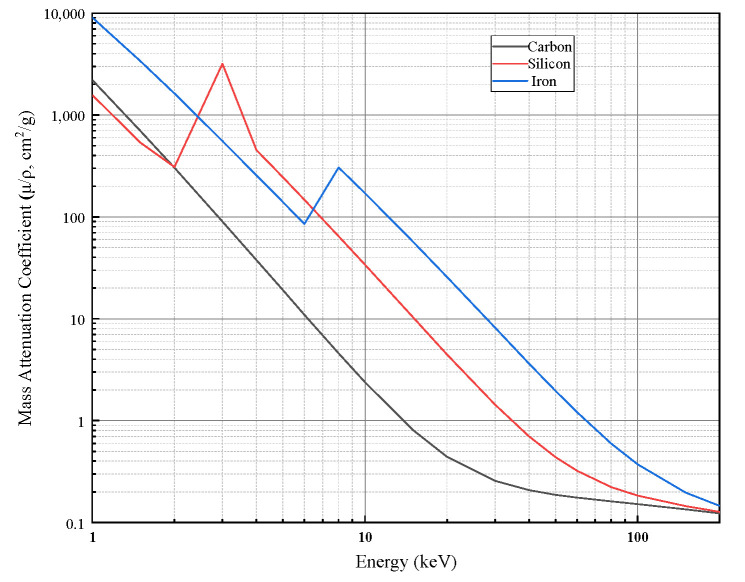
Mass attenuation coefficients of different materials as a function of photon energy.

(2) I=∫S(E)exp(−∫μ(E,r) dl)dE 

where S(E) denotes the source spectral distribution. This polychromatic nature gives rise to beam hardening: lower-energy photons are preferentially absorbed, causing the transmitted spectrum to shift toward higher energies (i.e., to become “harder”), which in turn biases the measured attenuation parameters away from their true values. The linear attenuation coefficient, μ(E), can be expressed as the sum of contributions from individual interaction mechanisms:

(3) $$\mu \left(E\right)=\rho \left[\tau \left(E\right)+\sigma_{\mathrm{inc}}\left(E\right)+\sigma_{\mathrm{coh}}\left(E\right)\right]$$

where ρ is the material density, and τ(E), $\sigma_{inc}(E)$, and $\sigma_{coh}(E)$ denote the mass attenuation coefficients associated with the photoelectric effect, Compton (incoherent) scattering, and Rayleigh (coherent) scattering, respectively. Mass attenuation coefficients of different materials as a function of photon energy are shown in Figure 1.

**Figure 2:**
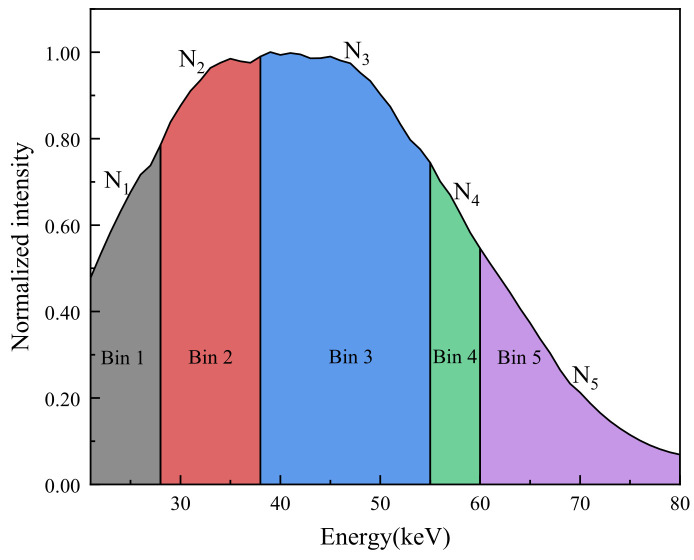
Schematic illustration of the threshold-based energy-bin configuration in the photon-counting detector (PCD). The band boundaries reflect the binning strategy used in this study, while the count levels in each bin are shown for illustration only.

Conventional energy-integrating detectors output an energy-weighted signal, thereby discarding the spectral information of incident photons. In contrast, photon-counting detectors (PCDs) perform event-by-event energy discrimination and counting, enabling the division of the incident spectrum into multiple energy bins (typically 4–8 bins). In practice, PCDs typically operate in energy spectrum scanning mode and multi-bin counting mode. The energy spectrum scanning mode acquires high-resolution continuous spectra by sweeping a narrow energy threshold window, which is crucial for system calibration, source spectrum analysis, and the construction of the detector response matrix (DRM). Conversely, the multi-bin counting mode simultaneously records photon counts across several predefined, broader energy windows. This mode enables highly efficient multi-spectral projection, making it the primary working mode for actual non-destructive testing and quantitative material characterization. Accordingly, in the multi-bin counting mode, the measured counts in the i-th energy bin, $N_{i}$, can be expressed as:

(4) $$N_{i}=\int_{E_{i}}^{E_{i+1}}S\left(E\right)\exp\left(-\int \mu \left(E,r\right)\;dl\right)R\left(E\right)dE$$

where R(E) denotes the detector energy-response function (ideally approximated by a rectangular energy window). As shown in Figure 2, based on the projection counts acquired in multiple energy bins, the equivalent linear attenuation coefficient for the i-th bin, $\mu_{t,i}$, can be further extracted, which represents the mean attenuation within that energy window:

(5) $$\mu_{t,i}=-\ln\left(\frac{N_{i}}{N_{i,0}}\right)/L$$

where $N_{i,0}$ denotes the air-scan (blank-field) counts in the i-th energy bin, and L is the X-ray path length through the specimen under investigation.

For multiphase composites, the effective atomic number $Z_{eff}$ can be introduced to characterize an equivalent elemental behavior. $Z_{eff}$ is commonly defined as the atomic number of a hypothetical single element whose attenuation characteristics (e.g., mass attenuation coefficient) match those of the composite under the same energy conditions. In the low-energy regime where the photoelectric effect dominates, the mass attenuation coefficient can be approximated as:

(6) $$\frac{\mu }{\rho }\approx C\left(E\right)Z^{\;k}$$

where C(E) is an energy-dependent coefficient and k is an empirical exponent, typically in the range of 3.0–4.0. For low-Z materials (Z<20), where the photoelectric contribution is relatively pronounced, the Mayneord empirical formulation with k=2.94 is commonly adopted:

(7) $$Z_{eff}={\left(\sum_{i}w_{i}Z_{i}^{2.94}\right)}^{1/2.94}$$

where $w_{i}$ denotes the electron-fraction weight of the i-th element. This formulation is well suited to low-Z composite systems involving elements such as carbon and silicon; the accuracy of the model for the C/C–SiC system will be further evaluated via simulations in subsequent sections. Based on multi-bin measurements, $Z_{eff}$ is commonly estimated using dual-energy or multi-energy decomposition schemes. A typical dual-energy approach assumes that the material can be represented as a mixture of two basis materials (e.g., carbon and SiC) [26,27]:

(8) $$\mu \left(E\right)=a_{1}f_{1}\left(E\right)+a_{2}f_{2}\left(E\right)$$

where $a_{1}$ and $a_{2}$ are the mass fractions of the two basis materials, and $f_{1}(E)$ and $f_{2}(E)$ are the known mass attenuation coefficients of the corresponding basis materials. The mass fractions $a_{1}$ and $a_{2}$ can be solved from the attenuation measurements $\mu_{t,1}$ and $\mu_{t,2}$ acquired in two energy bins, thereby enabling quantitative estimation of the constituent contents.

Multi-energy approaches can be extended to a larger number of energy bins, enabling further decoupling of density and $Z_{eff}$, or alternatively allowing direct matching to theoretical attenuation curves. In this work, a fine-grained, simulation-driven lookup-table strategy is adopted in subsequent sections to reduce reliance on empirical formulas and to improve quantitative characterization accuracy for complex multiphase systems.

### 2.2. Modeling and Correction of PCD Spectral Response Distortions

To obtain an effective atomic number ($Z_{eff}$) that faithfully reflects the intrinsic atomic properties of a material, hardware-induced spectral response distortions must be corrected in advance. In the 20–80 keV energy range, CdZnTe photon-counting detectors (PCDs) exhibit pronounced K-escape fluorescence, charge-sharing, and finite energy-resolution effects. These mechanisms cause the measured spectrum to deviate substantially from the ideal incident spectrum. Without proper correction, the resulting attenuation vector becomes systematically biased, which in turn compromises the accuracy of $Z_{eff}$ estimation.

This study constructs a detector response matrix (DRM) using Geant4-based Monte Carlo simulations and factorizes it as follows:

(9) $$R=R_{1}R_{2}$$

where $R_{1}$ denotes the K-escape and fluorescence-redistribution matrix, and $R_{2}$ represents the matrix accounting for charge sharing and finite energy resolution. The relationship between the measured spectrum and the ideal incident spectrum can be written as:

(10) $$S_{meas}=RS_{ideal}$$

The ideal incident spectrum, accounting for beam hardening, can be expressed as:

(11) $$S_{ideal}\left(E\right)\;=\;S_{0}\left(E\right)\;\exp\left(\;\;-\;\sum_{k}\mu_{k}\left(E\right)\;L_{k}\;\right)$$

**Figure 3:**
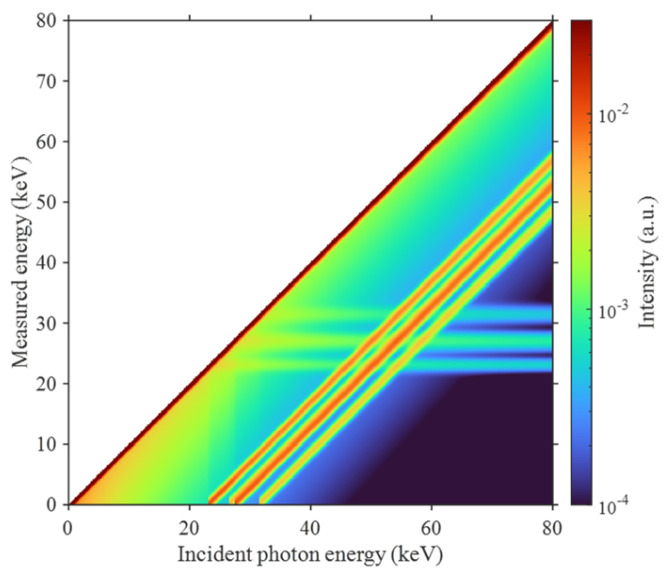
Heatmap of the detector response matrix (DRM).

To describe the detector-response distortion in a clearer way, the total response matrix was written as $R=R_{1}R_{2}$. Here, $R_{1}$ mainly describes fluorescence-escape-induced energy redistribution, whereas $R_{2}$ accounts for the additional count spreading and spectral broadening caused by charge sharing and finite energy resolution. In this study, this factorized form is introduced mainly for convenience in physical description and model construction, rather than as a separate theoretical contribution. The main advantage of this treatment is that different sources of detector distortion can be represented more clearly and then incorporated into the same correction framework. In practice, what matters is not the factorized form itself, but whether it helps improve the recovery of multispectral attenuation features and supports the subsequent constituent inversion. Therefore, in the present work, as shown in Figure 3, $R=R_{1}R_{2}$ should be understood as a practical way to organize detector-response modeling in the forward correction process.

In addition, beam hardening and detector-response distortion are coupled in the present photon−counting measurement process. If only beam hardening is corrected, the low-energy count redistribution caused by detector non-idealities will remain. If only detector-response distortion is considered, the spectral shift introduced by the polychromatic source will still affect the attenuation results. Therefore, both effects are treated jointly in the present model.

Let the measured count vector across n energy bins (with n=5 in this study) be $I={\left[I_{1},\cdot \cdot \cdot ,I_{n}\right]}^{T}\in R^{n}$, and let the corresponding forward prediction be $\hat{I}=RS$. Here, $R\in R^{n\times m}$ denotes the overall response matrix, factorized as $R=R_{1}R_{2}$, and $S\in R^{nm}$ is the ideal incident spectrum discretized on an energy grid, including the beam-hardening term. The iterative residual is defined as:

(12) $$\varepsilon =\frac{{\left\|I-\hat{I}\right\|}_{2}}{{\left\|I\right\|}_{2}}$$

Step 1. Initialization. Set $\mu^{\left(0\right)}=\mu_{meas}$.

An initial attenuation estimate is first assigned from the measured multispectral data. This initialization places the iteration close to the physically relevant solution domain and provides a stable starting point for the subsequent coupled correction. In this way, the effects of beam hardening and detector-response distortion are updated simultaneously within the same forward model, rather than being treated as two independent correction steps.

Step 2. Forward prediction. Given the current estimate $\mu^{\left(k\right)}$, compute the ideal spectrum $S_{ideal}^{\left(k\right)}$, and obtain the predicted spectrum by applying the response matrix:

(13) $$S_{pred}^{\left(k\right)}={RS}_{ideal}^{\left(k\right)}$$

Based on the current attenuation estimate, the corresponding ideal transmitted spectrum is first calculated by considering object attenuation under the polychromatic X-ray source. The predicted detector output is then obtained by applying the detector response matrix to the ideal spectrum. In this way, the forward prediction is generated under the same physical chain as the actual measurement, namely, object attenuation followed by detector-response distortion.

Step 3. Correction estimation via residual minimization. Estimate the correction Δμ by solving:

(14) $$\underset{∆\mu }{\min}{\left\|S_{meas}-R\left[S_{ideal}^{\left(k\right)}⊙exp\left(\;-\;∆\mu \cdot L\right)\right]\right\|}^{2}$$

The correction term is estimated by minimizing the mismatch between the measured count vector and the forward-predicted count vector. The exponential form in Equation (14) is used to keep the update consistent with the Beer–Lambert attenuation model. When the attenuation estimate is updated by Δμ, the transmitted spectrum changes multiplicatively as exp(−Δμ⋅L), rather than through an additive correction on the measured counts. In this way, the update remains physically consistent with polychromatic attenuation and the DRM-based forward model.

Step 4. Update and stopping criterion. Update the attenuation estimate as $\mu^{\left(k+1\right)}=\mu^{\left(k\right)}+∆\mu$ and iterate until the residual drops below 1%.

The attenuation estimate is updated using the correction term, and the iteration continues until the residual falls below the preset threshold of 1%. In the present study, this stopping criterion is used as a practical indicator that the measured and forward-predicted multi-bin counts have reached sufficient consistency within the unified physical model. We do not claim a general analytical proof of global uniqueness for the coupled solution in the present work. Instead, the practical stability of the algorithm is supported by the measured-data-based initialization, the residual-controlled iterative update, and the experimentally observed consistency of the corrected results across specimens with different thicknesses and constituent contents. Through this procedure, the recovered multispectral attenuation vector is obtained within a physically consistent correction framework, rather than through separated empirical compensation steps.

It should be noted that the objective of the above joint correction is not limited to spectrum restoration itself. In the present work, the corrected multispectral attenuation vector is used as an intermediate physical representation for subsequent constituent inversion in C/C–SiC composites. This point distinguishes the present framework from studies whose primary endpoint is detector-response analysis, spectrum unfolding, or $Z_{eff}$—oriented material identification. More importantly, detector-response distortion and beam-hardening effects are incorporated jointly into a unified forward modeling framework, rather than treated as two separable post-processing steps applied independently to the measured data. Therefore, standalone inversion results corresponding to “detector-response correction only” or “beam-hardening correction only” are not defined as physically self-consistent outputs within the present workflow.

### 2.3. Quantitative Characterization of Constituent Contents in C/C–SiC Composites

To achieve quantitative inversion of SiC mass fraction in C/C–SiC composites, a fine-grained theoretical database is constructed by taking SiC mass fraction as the key inversion parameter. The database covers the practical composition range from 0% to 100%, with a step size of 1%, so that the corrected attenuation features obtained from the joint correction can be directly linked to constituent-content inversion through quantitative matching. Accordingly, the present lookup-table strategy is most suitable for material systems whose constituent space can be reasonably parameterized in advance; for more open-ended scenarios, the database design and inversion space would need to be correspondingly expanded.

The material composition model simplifies the C/C–SiC composite into a three-phase mixture consisting of carbon fibers, a carbon matrix, and a SiC ceramic phase. Because the attenuation characteristics of the carbon fibers and the carbon matrix are similar, they are approximated as a single carbon phase and represented by graphitized carbon, while the SiC phase is represented by β − SiC. The densities are set to 2.0 g/cm^3^ for the carbon phase and 3.2 g/cm^3^ for the SiC phase. When the mass fraction of the SiC phase $\phi_{SiC}$ is treated as the variable parameter, the corresponding mass fraction of the carbon phase is $1-\phi_{SiC}$.

**Figure 4:**
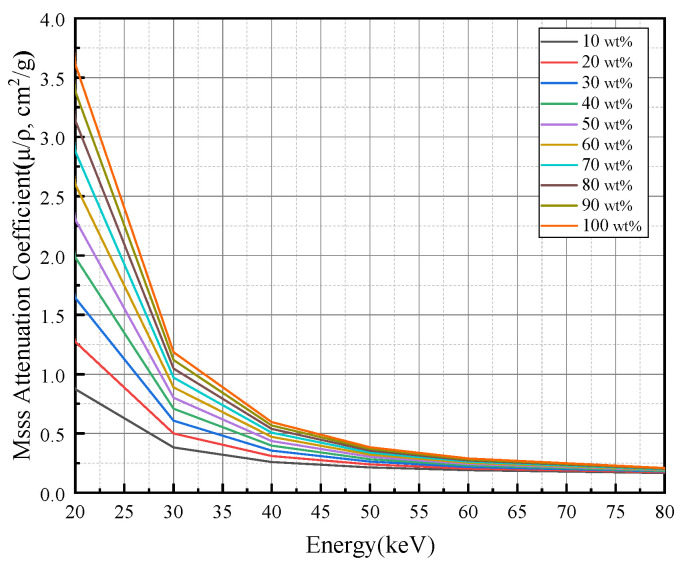
Theoretical mass attenuation coefficient curves of C/C–SiC composites at different SiC mass fractions.

The theoretical mass attenuation coefficients are obtained from the XCOM database. The mass attenuation coefficients of the carbon and SiC phases are queried over the energy range of 20–80 keV, and the mass attenuation coefficient of the mixture is then computed using mass-fraction weighting. The theoretical mass attenuation coefficient curves of carbon/carbon-silicon carbide (C/C–SiC) composites at different silicon carbide (SiC) mass fractions are shown in Figure 4.

(15) $${\left(\frac{\mu }{\rho }\right)}_{mix}\left(E\right)=\phi_{SiC}{\left(\frac{\mu }{\rho }\right)}_{SiC}\left(E\right)+\left(1-\phi_{SiC}\right){\left(\frac{\mu }{\rho }\right)}_{C}\left(E\right)$$

The ideal multi-bin projection data are generated by assuming an ideal photon-counting detector with no response distortions. Under this assumption, a forward projection is performed for a representative continuous spectrum S(E) at a tube voltage of 80 kVp, yielding the theoretical vector of equivalent linear attenuation coefficients for a given SiC mass fraction $\phi_{SiC}$,

(16) $$\mu_{\mathrm{theory}}\left(\phi_{\mathrm{SiC}}\right)=\left[\mu_{t,1},\;\mu_{t,2},\;\ldots ,\;\mu_{t,n}\right]$$

where n denotes the number of energy bins (n=5 in this study).

**Figure 5:**
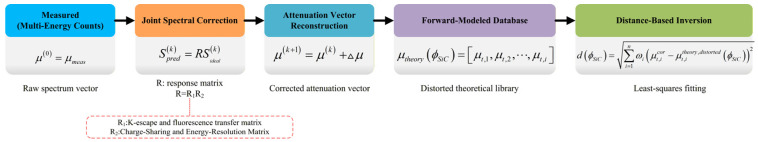
Flowchart of SiC mass-fraction inversion.

To ensure consistency with experimental measurements, detector-response distortions are explicitly taken into account. The theoretical attenuation coefficients are forward-mapped through the distortion matrix R established in Section 3 to generate a distorted attenuation database, which is subsequently used for direct lookup and matching. The SiC mass fraction in C/C–SiC composites is quantified using a lookup-table matching strategy, in which the experimentally measured attenuation vector is compared with database entries and the composition is determined by the minimum-distance criterion. The overall procedure is illustrated in Figure 5.

Experimental data are acquired using a photon-counting detector to obtain the raw counts $N_{i}$(i=1,…,n) in each energy bin and the corresponding air-scan counts $N_{i,0}$. The measured attenuation vector is then computed as

(17) $$\mu_{\mathrm{meas}}=\left[\mu_{t,1}^{\mathrm{meas}},\mu_{t,2}^{\mathrm{meas}},\ldots ,\mu_{t,n}^{\mathrm{meas}}\right]$$

A joint correction algorithm that accounts for both beam hardening and detector-response distortions is subsequently applied to obtain the corrected attenuation vector $\mu_{cor}$. For database matching, a weighted Euclidean distance was used:

(18) $$d\left(\phi_{\mathrm{SiC}}\right)=\sqrt{\sum_{i=1}^{n}w_{i}{\left(\mu_{t,i}^{\mathrm{cor}}-\mu_{t,i}^{\mathrm{theory},\mathrm{distorted}}\left(\phi_{\mathrm{SiC}}\right)\right)}^{2}}$$

The $\phi_{SiC}$ associated with the minimum distance is selected as the inversion result. After identifying the minimum-distance node, linear interpolation among the neighboring grid points was used to refine the estimated SiC mass fraction.

Because the present lookup table contains only a one-dimensional composition parameter and five multispectral bins, the matching cost is low and scales linearly with the number of database entries. For the current C/C–SiC inversion problem, the computational burden of the lookup step is therefore modest compared with the preceding correction step. Since the present database spans the full physically admissible SiC mass-fraction interval of 0–100% for the target C/C–SiC system, the inversion is confined to this predefined range. If the true material deviates from the assumed constituent model or lies outside the predefined database space, the result should be interpreted as a limitation of the assumed database model rather than as a valid extrapolated inversion.

## 3. Experiments and Validation

### 3.1. System Configuration

**Figure 6:**
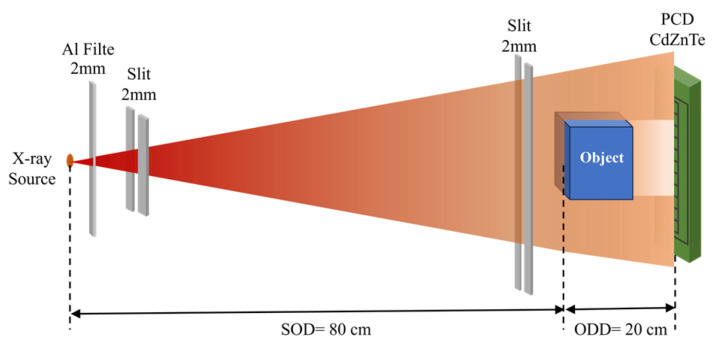
Schematic of the experimental system consisting of a microfocus X-ray source and a photon-counting detector.

A projection-imaging platform consisting of a microfocus X-ray source and a photon-counting CdZnTe detector was employed, as illustrated in Figure 6. The system mainly comprises an X-ray source and a photon-counting detector.

The source is a tungsten-target microfocus tube operated at 80 kVp and 300 μA. The emitted spectrum is a representative continuous spectrum with an effective energy range of 20–80 keV, and a 2 mm Al filter is placed at the output window for pre-hardening. The detector is a CdZnTe pixelated photon-counting array with a pixel size of 2 × 1 mm and 128 × 1 pixels. During the experiment, the PCD was utilized in both of its primary working modes to ensure data comprehensiveness and accuracy. Initially, the energy spectrum scanning mode was employed to measure the transmitted continuous spectra of the specimens, facilitating the observation of spectral distortions and guiding the optimal selection of energy thresholds. Subsequently, for the quantitative constituent inversion, the system was configured to the multi-bin counting mode. Five energy-bin thresholds were set at 20, 28, 38, 55, and 80 keV to divide the incident X-ray spectrum into discrete analytical windows. In addition, an Am-241 source was used to calibrate the detector threshold-to-energy relationship before quantitative measurements.

The source-to-object distance (SOD) was set to 80 cm, and the object-to-detector distance (ODD) was set to 20 cm. To restrict scatter and define the beam geometry, 2 mm-wide collimators were placed both at the X-ray source exit window and immediately upstream of the sample. This slit-collimated geometry was adopted to approximate a near-narrow-beam measurement condition, so that the experimental data remain consistent with the forward-model assumption used in the subsequent correction and inversion steps.

### 3.2. Samples and Materials

Experimental validation was conducted in two parts. First, an aluminum thickness-gradient specimen was used as a reference standard to evaluate the accuracy of the proposed detector-distortion correction algorithm. A high-purity aluminum step wedge was employed, with thicknesses of 3.33, 5.55, 7.4, 14.8, 25.95, 33.3, and 40.7 mm, and the atomic number of aluminum is Z=13. Second, C/C–SiC reference specimens with known SiC mass fractions were used to validate the accuracy of constituent-content inversion. The SiC content is expressed as the mass fraction $\phi_{SiC}$, which takes values of 7.74%, 14.37%, 20.11%, 25.12%, 29.55%, 33.48%, 45.62%, 47.99%, and 55.72%, respectively. The corresponding thicknesses of these specimens ranged from 21 mm to 35 mm (specifically 21, 22, 23, 24, 25, 26, 30, 31, and 35 mm). In addition, to evaluate measurement repeatability, the C/C–SiC specimens with SiC mass fractions of 7.74 wt% and 29.55 wt% were each measured 10 times under identical experimental conditions.

## 4. Results and Discussion

### 4.1. Verification of the Detector Response Matrix

**Figure 7:**
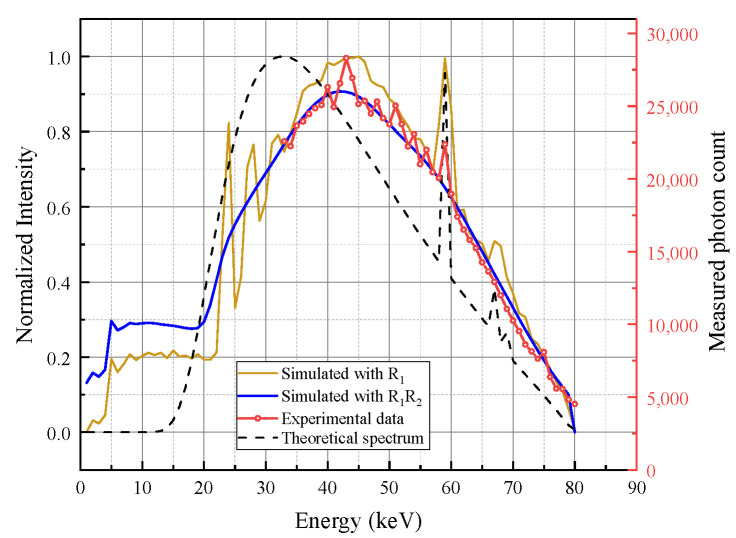
Spectral comparison of theoretical, DRM-simulated ($R_{1}$ and $R_{1}R_{2}$), and experimental data under blank-field conditions.

We validated the Geant4-simulated detector response matrix (DRM) under a blank-field condition. The baseline measurement was conducted using the 80 kVp microfocus X-ray source equipped with a 2 mm Al filter for pre-hardening, without any test specimen in the beam path. To demonstrate the step-by-step physical degradation of the spectrum, Figure 7 compares the idealized theoretical incident spectrum, the simulated spectra with partial ($R_{1}$) and full ($R_{1}R_{2}$) response matrices, and the experimental data.

As illustrated in Figure 7, the idealized theoretical 80 kVp spectrum (black dashed line) exhibits a concentrated energy distribution with sharp characteristic tungsten peaks. When only the K-escape and fluorescence redistribution matrix $R_{1}$ is applied (gold dashed line), distinct escape-related features appear and part of the photon counts is redistributed toward lower energies. However, the simulated spectrum still remains noticeably sharper than the experimental result and does not reproduce the overall spectral broadening observed in the real detector output. After further incorporating the charge-sharing and finite-energy-resolution matrix $R_{2}$, the full DRM-simulated spectrum ($R_{1}R_{2}$, blue solid line) exhibits a pronounced low-energy tail and a substantially broadened main peak, showing much better agreement with the measured spectrum (red line with circles). These results indicate that, although fluorescence escape contributes to the low-energy redistribution, explicit modeling of charge sharing and finite energy resolution is also necessary to reproduce the measured spectral distortion in the CdZnTe PCD.

Although the experimental spectrum exhibits an offset relative to the ideal theoretical spectrum, this mismatch is attributed mainly to the residual threshold-to-energy calibration offset of the detector system, rather than to a true shift of the tungsten characteristic X-ray energies. In the present detector, the threshold settings are implemented electronically as dimensionless threshold codes and must be converted to physical energies through calibration before use. Based on the Am-241 calibration result, the detector energy axis shows an offset relative to the theoretical spectrum. Under high photon flux, pulse pile-up may further broaden the peak shape and slightly affect the apparent centroid.

### 4.2. Evaluation of the Joint Correction Using an Aluminum Step Wedge

**Figure 8:**
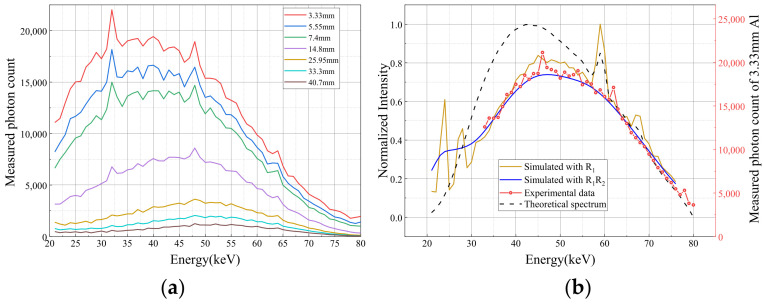
Spectral Scanning Results of Al. (**a**) Spectral scanning results for aluminum reference specimens with different thicknesses. (**b**) Comparison between simulated and experimental spectra for an 80 kVp source with a 2 mm Al filter.

The raw multi-bin count spectra acquired experimentally were processed using the proposed joint correction algorithm to obtain corrected equivalent attenuation coefficients for each energy bin. The corrected results were compared with theoretical reference values from the NIST XCOM database, as summarized in Figure 8. To ensure a consistent comparison with monoenergetic tabulations, an effective energy was computed for each energy bin using spectral weighting. Specifically, the effective energy of the k-th bin, $E_{eff,k}$, was defined through spectrum-and-response weighting. The experimentally derived attenuation coefficients are denoted as $\mu_{eff}$, which correspond to the monoenergetic linear attenuation coefficients tabulated by XCOM at $E_{eff,k}$.

(19) $$E_{eff,k}=\frac{\int E\;S\left(E\right)R_{k}\left(E\right)dE}{\int \;S\left(E\right)R_{k}\left(E\right)dE}$$

where S(E) denotes the X-ray source spectrum, and $R_{k}(E)$ represents the effective response of the k-th energy bin, incorporating both the energy-threshold settings and the detector response after the DRM is applied. In this study, the analysis primarily relies on the low-energy bin. For low-Z materials, the photoelectric effect dominates at low energies and the attenuation exhibits stronger Z-dependence, making the low-energy bin particularly sensitive to Z (or $Z_{eff}$). The effective energy associated with the selected low-energy bin is $E_{eff}=25.5\;\mathrm{keV}$. Before correction, the attenuation coefficient in the low-energy bin and the resulting $Z_{eff}$ estimates deviated from the theoretical values by approximately 30–40%, primarily due to the combined effects of beam hardening and K-escape-induced spectral distortions. After applying the proposed joint correction, the $Z_{eff}$ estimation accuracy improved to within ±0.6, corresponding to a relative deviation below 3%.

**Figure 9:**
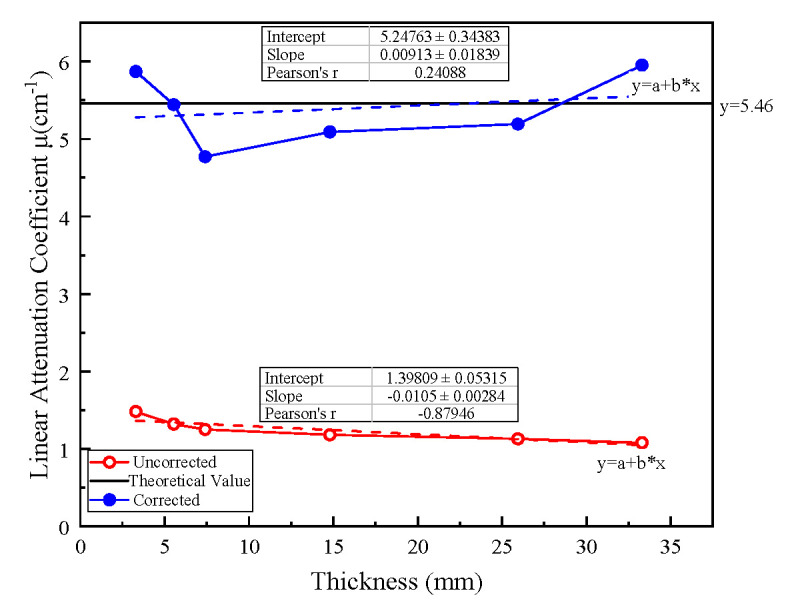
Comparison of the effective attenuation coefficients $\left(\mu_{eff}\right)\;$ of the aluminum step-wedge specimen before and after joint correction.

- Spectral Scanning Results;

As illustrated in Figure 8, the raw pulse height spectra (PHS) obtained from the aluminum step wedge exhibit significant spectral distortion. This phenomenon is primarily attributed to the beam-hardening effect, where the low-energy photons are preferentially absorbed as the sample thickness increases, leading to a shift in the mean energy of the transmitted spectrum. To suppress these systematic artifacts, the established detector response matrix (DRM) was applied within the iterative joint correction framework. By incorporating the $R_{1}\times R_{2}$ response model, the distorted counts in the low-energy bins—caused by charge-sharing and K-escape—were redistributed to their corresponding incident energies. The effectiveness of this restoration is quantitatively demonstrated in Figure 9, where the deviation in attenuation coefficients across different thicknesses is substantially minimized, ensuring the physical consistency required for subsequent $Z_{eff}$ inversion.

**Figure 10:**
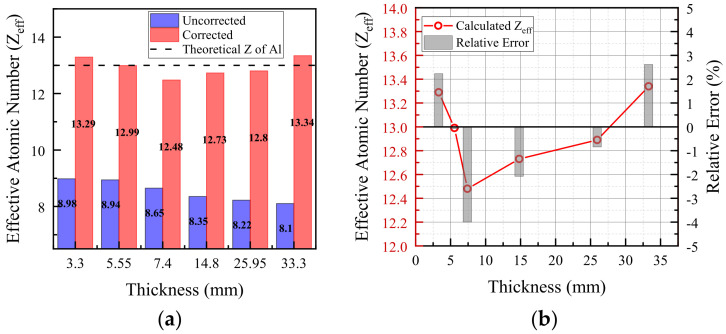
Estimated effective atomic number ($Z_{eff}$) for the aluminum reference specimen. (**a**) Comparison of $Z_{eff}$ estimates before and after joint correction across different thicknesses. (**b**) Calculated $Z_{eff}$ and corresponding relative errors for the corrected results.

- Energy-Bin-Resolved Results;

The attenuation coefficient was calculated as $\mu_{\mathrm{eff},k}=\frac{1}{L}ln\frac{I_{0,k}}{I_{k}}$, where $I_{0,k}$ and $I_{k}$ denote the air-scan and transmitted counts in the k-th energy bin, respectively, and L is the sample thickness. Here, the uncorrected result was obtained by directly calculating $\mu_{eff,k}$ from the experimental multi-bin data, while the corrected result was obtained after applying the proposed joint correction. Prior to correction, $\mu_{eff}$ showed a strong negative dependence on thickness, revealing a systematic bias caused by beam hardening and detector-response distortions. After applying the proposed joint correction, the thickness-dependent slope decreased by 13.1%, and the cross-thickness dispersion, quantified by the coefficient of variation (CV), was reduced by 27.0%, indicating a markedly improved thickness consistency. These results demonstrate that the proposed method not only improves the internal consistency of the corrected attenuation coefficients, but also provides a clear quantitative advantage over the conventional direct calculation based on uncorrected multi-bin data. The results of the estimated effective atomic number ($Z_{eff}$) for the aluminum reference specimen are shown in Figure 10.

### 4.3. Quantitative Constituent Inversion and Error Analysis for C/C–SiC Samples

**Figure 11:**
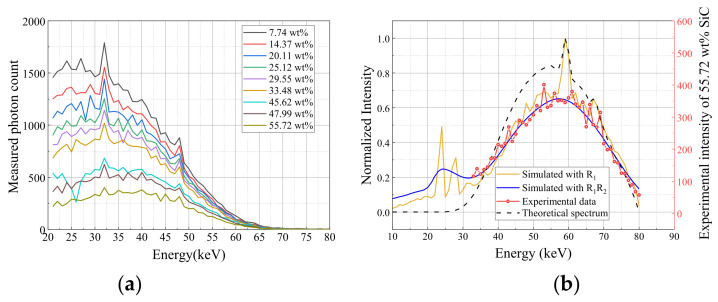
Spectral Scanning Results of SiC. (**a**) Measurement results for C/C–SiC specimens with different SiC mass fractions. (**b**) Comparison between simulated and experimental spectra for a C/C–SiC specimen of 25.12 wt% SiC.

- Spectral Scanning Results;

Following the validation on the aluminum reference, the joint correction framework was applied to the complex C/C–SiC multi-phase system. As shown in Figure 11a, the measured photon counts decrease significantly with increasing SiC mass fraction ($\phi_{SiC}$), reflecting the higher macro-attenuation of the silicon-containing phase compared to the carbon matrix. However, raw spectral data remain coupled with detector artifacts, as evidenced by the discrepancy between the theoretical 80 kVp spectrum and the experimental measurements in Figure 11b.

**Figure 12:**
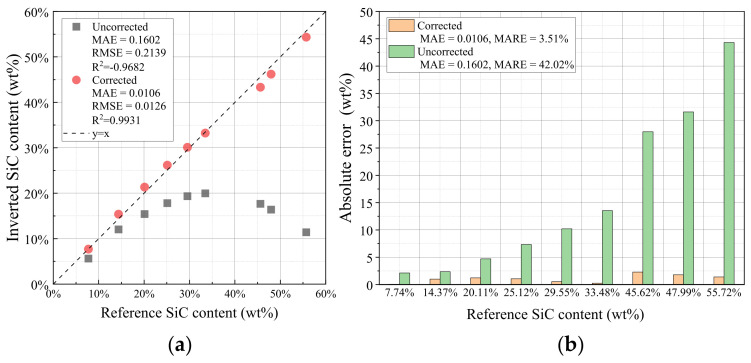
Inversion results of the SiC mass fraction for C/C–SiC specimens. (**a**) Correlation between the inverted and reference SiC mass fractions. (**b**) Absolute error distribution of the inverted SiC mass fractions.

- Energy-Bin-Resolved Results;

Using the lookup-table-based inversion approach, the corrected attenuation vectors were matched to entries in the fine-grained database to determine the constituent contents. The quantitative inversion results, summarized in Table 1, yield a Mean Absolute Error (MAE) of 1.06 wt%, a Mean Absolute Relative Error (MARE) of 3.51%. Furthermore, the inverted values exhibit a strong linear correlation with the reference values, with an RMSE of 1.26 wt% and an $R^{2}$ of 0.98 (Figure 12).

For comparison, the uncorrected direct-decomposition results were also evaluated using the same specimen set. Based on the data in Table 1, the uncorrected results yield an MAE of 16.02 wt%, an RMSE of 21.39 wt%, and a MARE of 42.02%. After joint correction, these values are reduced to 1.06 wt%, 1.26 wt%, and 3.51%, respectively, corresponding to improvement percentages of 93.35%, 94.09%, and 91.65%. These results show that the proposed method provides a clear quantitative improvement over the conventional direct decomposition based on uncorrected multi-bin measurements.

**Figure 13:**
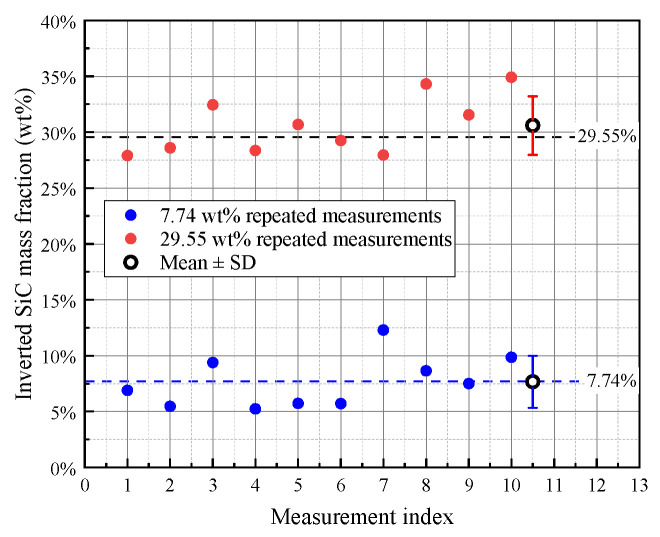
Repeatability evaluation of SiC mass-fraction inversion for representative C/C–SiC specimens. The scatter points show the results of 10 repeated measurements for the specimens with reference SiC mass fractions of 7.74 wt% and 29.55 wt%, respectively. The larger open circles and error bars denote the mean ± SD, and the dashed horizontal lines indicate the corresponding reference values.

To further evaluate measurement repeatability, two representative C/C–SiC specimens with SiC mass fractions of 7.74 wt% and 29.55 wt% were each measured 10 times under identical conditions. The corresponding repeated inversion results are shown in Figure 13, and the statistical summary is listed in Table 2. For the 7.74 wt% specimen, the repeated measurements yielded an average inverted value of 7.68 ± 2.33 wt%, with a Type A standard uncertainty of 0.74 wt% and a 95% confidence interval of 6.01–9.35 wt%. For the 29.55 wt% specimen, the repeated measurements yielded an average inverted value of 30.60 ± 2.62 wt%, with a Type A standard uncertainty of 0.83 wt% and a 95% confidence interval of 28.72–32.47 wt%. These results provide repeatability-based statistical information in addition to the specimen-wise accuracy metrics reported above. They also indicate that the inversion is more stable for the medium-SiC specimen, whereas the low-SiC specimen is more sensitive to measurement fluctuation, although its mean inverted value remains close to the reference value.

### 4.4. Discussion

1. Physical Advantages of $Z_{eff}$ and Impact of Joint Correction.

The experimental results from the aluminum step-wedge and C/C–SiC specimens demonstrate that the proposed joint correction significantly stabilizes the extracted physical parameters relative to the conventional direct calculation from uncorrected multi-bin data. For complex multiphase systems, the transition from analyzing the linear attenuation coefficient μ to the effective atomic number $Z_{eff}$ is pivotal, offering two primary physical advantages. To successfully decouple composition from density and porosity in heterogeneous materials like C/C–SiC, it is essential to overcome the linear attenuation coefficient μ’s strong dependence on the bulk density ρ. By utilizing the effective atomic number $Z_{eff}$, which solely reflects the atomic composition of the substance, the characterization remains inherently independent of the sample’s porosity or looseness. This intrinsic property effectively eliminates the interference of porosity on constituent determination, thereby ensuring that variations in measured counts are accurately mapped to chemical changes rather than structural voids. It should be noted that the main purpose of this work is not to introduce a new universal DRM formulation, but to build a quantitative characterization framework for C/C–SiC composites under realistic multispectral photon-counting X-ray detection conditions. In this framework, detector-response modeling, polychromatic beam-hardening correction, and fine-grained constituent-database inversion are incorporated into a unified procedure, so that constituent quantification can be carried out in a way that is more consistent with the actual measurement chain.

The suppression of thickness-dependent bias is another critical advantage, as conventional X-ray methods often suffer from systematic deviations as sample thickness increases, primarily due to coupled beam-hardening and detector-response distortions. In the present work, these two effects are incorporated simultaneously into the forward system-matched simulation chain, and therefore a strictly independent BHC-only intermediate result is not explicitly separated from the joint-correction workflow. Accordingly, the quantitative comparison is established between the uncorrected direct calculation/direct decomposition results and the jointly corrected results. By leveraging the multi-energy binning characteristics of photon-counting detectors (PCDs) and the proposed joint correction, the $Z_{eff}$ obtained through multi-spectral data can effectively offset these measurement biases. As shown in the aluminum step-wedge experiment (Figure 10), without correction, the attenuation coefficients exhibited large deviations of approximately 30–40% and a pronounced nonlinear dependence on thickness. After applying the joint correction, the thickness-dependent slope was markedly reduced, and the $Z_{eff}$ achieved an accuracy within ±0.6 (relative deviation < 3%). This result indicates that the proposed method improves not only the internal consistency of corrected spectra, but also the robustness of attenuation-based parameter extraction relative to the conventional direct calculation from uncorrected multi-bin measurements.

2. Inversion Accuracy and Statistical Analysis for C/C–SiC Constituents

By matching the jointly corrected multi-bin attenuation vector to the fine-grained database, the SiC mass fractions of 9 C/C–SiC reference specimens were successfully inverted. Compared with the uncorrected direct-decomposition results, the jointly corrected results show a consistently better agreement with the reference values. For the full specimen set, the uncorrected inversion yields an MAE of 16.02 wt%, an RMSE of 21.39 wt%, and a MARE of 42.02%, whereas the corresponding values after joint correction are reduced to 1.06 wt%, 1.26 wt%, and 3.51%, respectively. This comparison confirms that the proposed correction-and-inversion workflow improves not only the consistency of the recovered attenuation features, but also the final constituent-quantification accuracy.

In low-Z composite systems, a single $Z_{eff}$ value essentially compresses multi-energy information into a scalar descriptor, making it susceptible to measurement noise and pixel-to-pixel variability. In contrast, the multi-bin attenuation vector matching adopted in this work preserves the multidimensional attenuation signatures that differentiate the carbon phase from the SiC phase across energy windows. The inherent density-independence of the $Z_{eff}$ logic, combined with this vector-based matching, effectively exploits the redundancy across energy dimensions to maintain robust inversion performance. This approach successfully circumvents the density fluctuations typical of complex composite manufacturing processes.

3. Error Sources and Uncertainty Analysis

Although the proposed joint correction significantly improves the quantitative characterization results, a small residual error is still present. From the current experiments, the main sources can be understood as follows. First, the raw PCD counts obey Poisson statistics, and the relatively narrow energy windows limit the effective counts in each bin, so statistical fluctuation cannot be completely avoided. Second, the measured spectra show an offset relative to the theoretical prediction. The dominant reason is the residual threshold-to-energy calibration offset of the detector system. Since the detector thresholds are implemented electronically as dimensionless threshold codes, a calibration step is required to establish the corresponding physical energy axis. In the present study, this calibration was checked using an Am-241 source. In addition, pulse pile-up under high photon flux may further broaden the peak shape and slightly influence the apparent centroid. By contrast, tube aging or additional inherent filtration mainly affects the continuum background and relative spectral weighting, rather than the characteristic-line energy itself. Third, for thicker specimens, the corrected attenuation coefficients remain slightly higher than the theoretical values. This is mainly attributed to the increasing probability of multiple scattering. Even after beam-hardening correction, some scattered photons may still be misclassified into lower energy bins, producing a mild build-up effect. This residual behavior is also consistent with the modeling boundary of the present study, because photons scattered inside the object and subsequently reaching the detector were not explicitly modeled as an independent transport term, but were instead suppressed experimentally by slit collimation.

A strict one-factor-at-a-time separation of all error sources is not available in the present study, but the current data still allow a basic quantitative interpretation of the residual error. Repeated measurements on two representative C/C–SiC specimens were used to estimate the short-term random fluctuation of the inversion results. For the 7.74 wt% and 29.55 wt% specimens, the repeated scans gave Type A standard uncertainties of 0.74 wt% and 0.83 wt%, respectively, together with the corresponding RSD values and 95% confidence intervals. These values mainly reflect the combined influence of counting statistics and short-term system fluctuation under identical measurement conditions. In addition, the difference between the repeated-measurement mean and the reference value provides a practical indication of the remaining systematic component. In the present results, the low-SiC specimen remains close to the reference value, whereas the medium-SiC specimen shows a modest positive bias, suggesting that residual model mismatch and spectral deviation still contribute to the final inversion error.

For the full set of 9 C/C–SiC reference specimens, the corrected inversion yields an MAE of 1.06 wt% and an RMSE of 1.26 wt%. Compared with the repeatability-based uncertainty obtained from repeated scans, this suggests that the final residual error is not caused by counting fluctuation alone, but also contains contributions from forward-model mismatch, spectral shift, and scattering-related deviation, especially for thicker specimens. Overall, the joint correction reduces the maximum relative error in $Z_{eff}$ from more than 30% in the uncorrected case to below 4%. At the present stage, the uncertainty analysis mainly provides a repeatability-based Type A evaluation. A more complete combined uncertainty budget, including long-term drift, environmental variation, and other systematic contributions, still needs further study.

4. Generalization and applicability of the present framework

The present framework is not intrinsically limited to aluminum or low-Z C/C–SiC composites, because its core structure is based on a unified forward model that jointly incorporates multispectral attenuation, beam hardening, and detector-response distortion. In this sense, the method has the potential to be extended to broader material systems. However, the current implementation is mainly developed and validated for low-Z, multiphase C/C–SiC composites, where the constituent space can be reasonably parameterized in advance. For higher-Z or more complex heterogeneous materials, stronger fluorescence redistribution, K-edge-related spectral discontinuities, scattering effects, and detector nonlinearities may become more significant, and the forward model, calibration, and database design would need further extension and validation. In addition, because the present inversion strategy relies on a fine-grained, system-matched database and prior parameterization of the constituent space, it is particularly suitable for controlled industrial scenarios, but less directly transferable to more open-ended applications such as broader medical imaging tasks.

The present lookup-table inversion is computationally lightweight for the current one-parameter C/C–SiC problem, but its extension to higher-dimensional constituent spaces would require a corresponding increase in database size and therefore further optimization of database design and search efficiency. Moreover, the present study was validated under a slit-collimated photon-counting projection geometry rather than a flat-panel broad-beam imaging condition. Therefore, scatter-dominated area-imaging scenarios with strong thickness variation and edge-related effects would require additional dedicated modeling and validation.

## 5. Conclusions

This study proposes a quantitative multispectral characterization framework for C/C–SiC composites based on photon-counting X-ray detection. By incorporating detector-response distortion and polychromatic beam-hardening effects into a system-matched processing chain, the method establishes a complete workflow from detector-response modeling and joint correction to multispectral feature recovery and constituent-content inversion.

Validation with the aluminum step wedge showed that the proposed method effectively suppresses thickness-dependent bias in the attenuation parameters. Experiments on C/C–SiC reference specimens further demonstrated good accuracy and stability in SiC mass-fraction inversion, indicating that the method improves both the consistency of the attenuation features and the reliability of the subsequent constituent characterization.

It should be noted that the present work mainly focuses on low-*Z*, multiphase C/C–SiC composites, where thickness variation and porosity-related perturbations significantly affect attenuation-based characterization. Moreover, because the present inversion strategy relies on a fine-grained, system-matched database and prior parameterization of the constituent space, the current implementation is particularly suitable for controlled engineering material systems rather than open-ended composition-identification tasks. In this sense, the main contribution of this study is not the proposal of a new universal DRM formulation, but the establishment of a physically consistent closed-loop framework for quantitative constituent characterization under realistic multispectral photon-counting X-ray detection conditions. Further work is still needed before the present framework can be extended to broader imaging scenarios involving stronger scatter collection, thickness variation, and edge-related effects. In particular, such extensions would require dedicated scatter-aware forward modeling and separate experimental validation, and are beyond the scope of the present study.

## Figures and Tables

**Table 1:** Comparison of constituent inversion results obtained by the conventional direct decomposition and the proposed joint correction method for C/C–SiC specimens.

Sample ID	$\mathrm{Reference}\;\phi_{SiC}$ (wt%)	$\mathrm{Corrected}\;Z_{eff}$	$\mathrm{Corrected}\;\phi_{SiC}$ (wt%)	$\mathrm{Uncorrected}\;Z_{eff}$	$\mathrm{Uncorrected}\;\phi_{SiC}$ (wt%)
1	7.74%	6.751059	7.70%	6.704353	5.61%
2	14.37%	7.365008	15.37%	7.364907	12.01%
3	20.11%	7.781902	21.33%	7.672371	15.40%
4	25.12%	8.091197	26.18%	7.875304	17.79%
5	29.55%	8.324824	30.09%	8.001264	19.34%
6	33.48%	8.504527	33.24%	8.050120	19.95%
7	45.62%	9.034939	43.33%	7.863465	17.65%
8	47.99%	9.174751	46.19%	7.757041	16.39%
9	55.72%	9.551826	54.33%	7.307104	11.41%

**Table 2:** Repeatability statistics of SiC mass-fraction inversion for representative C/C–SiC specimens based on 10 repeated measurements.

Sample ID	$\mathrm{Reference}\;\phi_{SiC}$ (wt%)	*n*	Inverted Result (Mean ± SD, wt%)	RSD (%)	$\mathrm{Type}\;A\;\mathrm{Uncertainty}\;u_{A}$(wt%)	95% CI (wt%)
1	7.74%	10	7.68 ± 2.33%	30.41%	0.74%	6.01–9.35%
5	29.55%	10	30.60 ± 2.62%	8.56%	0.83%	28.72–32.47%

## Data Availability

Data are contained within the article.

## Abbreviations

The following abbreviations are used in this manuscript:

ASICs	Application-Specific Integrated Circuits
C/C–SiC	Carbon/Carbon–Silicon Carbide
CT	Computed Tomography
CV	Coefficient of variation
CVI	Chemical Vapor Infiltration
DRM	Detector response matrix
EDS	Energy-dispersive spectroscopy
MAE	Mean Absolute Error
MARE	Mean Absolute Relative Error
NDT	Non-destructive testing
NIST	National Institute of Standards and Technology
ODD	Source-to-detector distance
PCD	Photon-counting detector
PHS	Pulse height spectra
PIP	Precursor Infiltration and Pyrolysis
RMI	Reactive Melt Infiltration
RMSE	Root Mean Square Error
SEM	Scanning electron microscopy
SOD	Source-to-object distance
TGA	Thermogravimetric analysis
THz-TDS	Terahertz time-domain spectroscopy
XCOM	X-ray Attenuation Database
XRD	X-ray diffraction
